# Experimental evidence of life history trade-offs during ultra-endurance physical activity

**DOI:** 10.1017/ehs.2026.10040

**Published:** 2026-03-06

**Authors:** Daniel P. Longman, Alison Murray, Emily L. Brown, Courtney Lewis, Richard M. Millis, Tomasz J. Nowak, Krizia-Ivana T. Udquim, Michael P. Muehlenbein, Jonathan C.K. Wells, Jay T. Stock

**Affiliations:** 1 School of Sport, Exercise and Health Sciences, Loughborough University, Loughborough, UK; 2 Department of Anthropology, University of Victoria, British Columbia, Canada; 3 Centre for Academic Primary Care, Population Health Sciences, University of Bristol Medical School, Bristol, UK; 4 Department of Pathophysiology, American University of Antigua, St. Johns, Antigua; 5 Department of Anthropology, Baylor University, Waco, TX, USA; 6 Department of Medicine, Inova Fairfax Medical Campus, Falls Church, VA, USA; 7 Childhood Nutrition Research Centre, Population Policy and Practice Department, UCL Great Ormond Street Institute of Child Health, London, UK; 8 Department of Anthropology, University of Western Ontario, London, ON, Canada

**Keywords:** life history theory, trade-offs, phenotypic plasticity

## Abstract

Life history theory seeks to understand how organisms distribute energy between physiological functions across the life course. A central assumption is that energy allocation involves ‘trade-offs’ between competing functions relating to defence, maintenance, reproduction, and growth. Constraints on human energy expenditure may produce trade-offs during energetic stress, affecting functions critical for homeostasis, survival, and reproduction. While there is some evidence for binary trade-offs between two functions in humans, no studies have tested physiological resource prioritisation across multiple functions under energetic constraint. This study empirically assessed multiple human life history trade-offs and the proximate biological mechanisms underpinning them. We recruited 147 ultra-endurance athletes (107 male, 40 female) participating in four environmentally diverse multiday ultramarathons and one multiweek ocean rowing event. The severe energetic demands of these competitions provide a valuable opportunity to provoke and observe detectable trade-offs. We found evidence of trade-offs across multiple functions. Specifically, investment in defence (as indexed by immune biomarkers) was broadly prioritised relative to investment in storage, reproduction and maintenance. Our results enhance current understanding of the role of phenotypic plasticity in human adaptability and have implications for athlete health and performance as well as the emerging discipline of evolutionary public health.

## Graphical abstract

**Figure:**
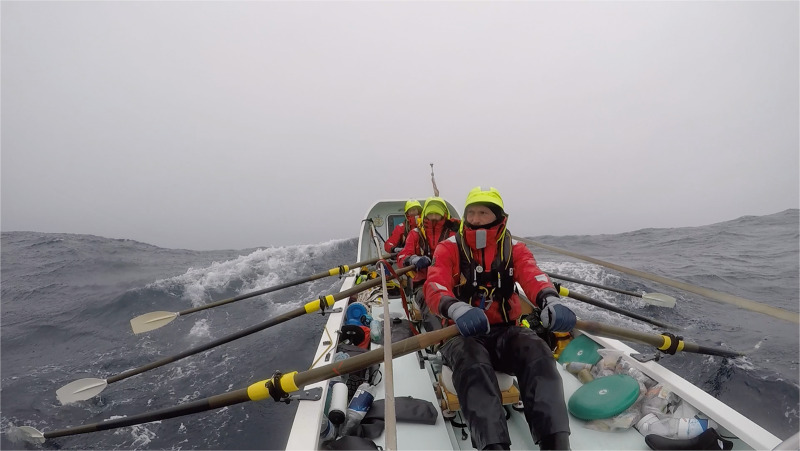

Significance statement

A core principle of life history theory is the existence of trade-offs in energy allocation between competing biological processes when available energy is scarce. However, there is limited experimental evidence that trade-offs in resource allocation – a cornerstone of life history theory – exist in humans. Here, we demonstrate evidence suggestive of the prioritisation of processes relating to defence at the expense of storage, reproduction, and homeostatic maintenance in a cohort of 147 energetically stressed ultra-endurance athletes. This evidence, which supports the role of trade-offs in our species, has interdisciplinary implications for the fields of anthropology, evolutionary public health and sports and exercise science.

## 1. Introduction

Life history theory is a branch of evolutionary theory that seeks to characterise the competitive allocation of limited resources between physiological functions (Leonard, 2012; Leonard & Ulijaszek, 2002; Saxne & Heinegard, 1992; Stearns, 1989; Ulijaszek, 1995; Wells et al., 2017; Zera & Harshman, 2001) optimal allocation pattern is determined by the individual’s life stage, sex, and environment (Holliday, 1986; Kramer et al., 2009; McDade et al., 2008), and is achieved in a large part through phenotypic plasticity (Hill & Hurtado, 1996). Due to its potential to improve current understanding of human health and behaviour, there is increasing interdisciplinary interest across the human sciences in applying the conceptual framework provided by life history theory in empirical research (Sear, 2020).

A fundamental assumption of life history theory is the existence of trade-offs between competing functions relating to defence, storage, reproduction, maintenance, and growth during periods of energetic stress (Bronson, 1991; Hill & Kaplan, 1999; Reznick et al., 2000; Stearns, 1992). Under such conditions, limited resources are predicted to be preferentially allocated to biological functions conferring the greatest benefit to evolutionary fitness at the expense of less essential functions. In non-human animals, experimental work with model systems such as insects, lizards and birds has provided compelling evidence for trade-offs and their implications for fitness (Zera & Harshman, 2001).

Although trade-offs are a cornerstone of life history theory and widely theorised, empirical evidence for their existence in our own species remains limited (Bolund, 2020; Flatt & Heyland, 2011; Sear, 2020) and the proximate mechanisms that mediate such trade-offs are poorly understood (Flatt & Heyland, 2011). The few studies that have demonstrated trade-offs in humans have evaluated binary covariation between pairs of life history functions. For example, Urlacher and colleagues reported evidence of a trade-off between immune function and growth in Shuar forager-horticulturalist children in Amazonian Ecuador. Under conditions of limited dietary energy intake and high rates of infectious and parasitic disease, children with greater immune activity tended to be shorter than their peers, indicating growth suppression in favour of defence (Urlacher et al., 2018).

This scarcity of evidence reflects the challenges associated with observing trade-offs in humans. Unlike researchers working with non-human species, who can experimentally manipulate environmental conditions to test life history hypotheses, human studies are constrained by stronger ethical limitations (Sear, 2020). In parallel, population-level observational data often mask trade-offs occurring at the individual level (Careau & Wilson, 2017), making it difficult to detect the negative covariance expected between traits in competition with each other (Cody, 1966; Glazier, 2000). Mechanistically, this masking effect may be driven, at least in part, by inter-individual differences in phenotypic quality (Wilson et al., 2014) and by the tendency for variation in resource acquisition to exceed variation in resource allocation between competing functions (van Noordwijk & de Jong, 1986). These challenges are exemplified by the persistent lack of clear evidence for the long-theorised trade-off between reproduction and survival (Bolund, 2020; Sear, 2020).

Here, we address these limitations by using ultramarathon athletes as a quasi-experimental model for examining patterns of energy allocation during energetic stress in the field (Longman et al., 2020). Ultramarathons impose substantial physiological demands, typically resulting in a negative energy balance that pushes physiological and cognitive systems to the limits of adaptive plasticity (Knechtle & Bircher, 2005; Knechtle et al., 2005). These conditions reduce the masking effects of inter-individual variation in phenotypic quality and resource acquisition, thereby increasing the likelihood of detecting life history trade-offs. Previous research using this approach identified acute binary trade-offs between immune defence and reproductive function biomarkers, with a reallocation of energetic investment towards immune function during a 100-mile running race (Longman et al., 2017). However, this study was limited in scope, focusing on only two life history functions, a relatively small sample size (ranging from 34 to 66 depending on the outcome) and male athletes.

In this study, we extend this approach to assess multivariate life history trade-offs in humans and to examine patterns of physiological resource prioritisation across multiple functions under conditions of energetic stress. We do so by (a) incorporating a broader suite of biomarkers spanning four life history functions; (b) recruiting a larger sample of ultra-endurance athletes (*n* = 147); and (c) increasing the number of females by selecting races with higher rates of female participation (*n* = 40). Participants were drawn from five environmentally diverse ultra-endurance events: four multiday ultramarathons (*n* = 111; 78 m, 33 f) and one multiweek ocean rowing competition (*n* = 36; 29 m, 7 f), Table 1.

**Table 1.** Biomarkers indexing energetic stress and life history functions

Function	Biomarker	Justification and interpretation
Energetic stress	Body mass	Reflects the net balance between energy intake and expenditure. A decrease from pre- to post-race indicate acute energetic stress and negative energy balance.
	Salivary cortisol	A steroid hormone produced by the adrenal glands in response to stress (Selye, 1946). An increase from pre- to post-race signals activation of the stress response and mobilisation of energy to meet acute demands.
Defence	Interleukin-6 (IL- 6)	A pleiotropic cytokine involved in immune and metabolic regulation, commonly used as a pro-inflammatory marker. Elevated levels demonstrate activation of immune and inflammatory pathways (Uciechowski & Dempke, 2020).
	Bacteria killing assay (BKA)	Assesses the ability of multiple components of serum to kill and inhibit bacterial growth *ex vivo* (Muehlenbein et al., 2011). Increases indicate stronger innate immune activity *ex vivo*.
	Haemolytic complement assay (HCA)	Measures the ability of the complement system to lyse red blood cells (Prall & Muehlenbein, 2014). Increased activity reflects stronger innate immune activity and therefore greater investment in immune function.
Storage	Fat mass index	Quantifies adipose tissue stores via bioelectrical impedance. Adiposity stores energy and supports female reproductive effort (Wells, 2010a, Wells, 2010b, 2018; Wells et al., 2017). Lower values signal reduced energy reserves available for future reproduction or survival.
	Leptin	A peptide hormone produced by adipose cells that regulates appetite and metabolism. Lower levels signal reduced energy stores and diminish capacity to support female reproductive function (Friedman, 2019; Frisch, 1987).
Reproduction	Testosterone	A predominantly male sex hormone that also circulates at lower levels in females, in whom it also contributes to reproductive function (Davis & Wahlin-Jacobsen, 2015; Muehlenbein & Bribiescas, 2005). Lower levels demonstrate reduced investment in reproductive effort for males.
	Oestradiol	A form of oestrogen and a predominantly female sex hormone involved in regulation of the menstrual cycle and development of female reproductive tissues (Hau, 2007). Decreases reflect reduced fecundability.
	Fat mass index	Quantifies adipose tissue stores via bioelectrical impedance. Adiposity stores energy and supports female reproductive effort (Wells, 2010a, Wells, 2010b, 2018; Wells et al., 2017). Lower values signal reduced energy reserves available for future reproduction or survival.
	Leptin	A peptide hormone produced by adipose cells that regulates appetite and metabolism. Lower levels signal reduced energy stores and diminish capacity to support female reproductive function (Friedman, 2019; Frisch, 1987).
Maintenance	Myoglobin	A protein in muscle cells that binds and stores oxygen. Elevated circulating levels signal skeletal muscle damage requiring maintenance (Brancaccio et al., 2010).
	Malondialdehyde (MDA)	A marker of lipid peroxidation and oxidative stress caused by free radicals and reactive oxygen species (Del Rio et al., 2005). Higher levels indicate greater cellular damage requiring maintenance.
	Total antioxidant capacity (TAC)	Reflects the overall ability of the body to neutralise free radicals and oxidative stress (Fraga et al., 2014). Higher values signal greater antioxidant capacity, reflecting greater investment in maintenance.
	Cartilage oligomeric matric protein (COMP)	A protein in cartilage and other connective tissues and serves as a marker of cartilage turnover (Saxne & Heinegard, 1992). Elevated levels demonstrate increased tissue remodelling and damage requiring maintenance.

Potential trade-offs between life history functions were assessed via biomarkers representing defence, maintenance, storage and reproduction. Measurements were taken at two time points: baseline (1–2 days prior to the race) and post-race, either 60 minutes after completion (the Finland Spain ultramarathons and the Atlantic Ocean rowing competition) or one day after completion at the same time of day as baseline testing (the Peru and Nepal ultramarathons). While biomarkers were selected to reflect energetic investment in specific life history functions, we acknowledge that the biomarkers selected have pleiotropic roles in human physiology, complicating their exclusive attribution to a single life history function.

Energetic stress was assessed via body mass and salivary cortisol. Defence was indexed by interleukin-6 (IL-6), a bacteria killing assay (BKA), and a haemolytic complement assay (HCA). Storage was assessed through fat mass index (FMI) and leptin. Reproductive effort in males was represented by testosterone and in females by oestradiol, FMI, leptin and testosterone. Maintenance was represented by markers of tissue damage and oxidative stress: myoglobin, malondialdehyde (MDA), total antioxidant capacity (TAC) and cartilage oligomeric matrix protein (COMP; see Table 1).

There is no ideal ‘model’ for the typical daily activity patterns of ancestral human populations, and neither contemporary ultra-endurance athletes nor hunter-gatherers serve as direct analogues. While there is some evidence for high levels of mobility in ancestral groups (Shaw & Stock, 2013), such patterns may not have been universal. Consistent with this variability, observed mobility among contemporary hunter-gatherers spans a wide range. For example, Hadza hunter-gatherers have been reported to cover between ∼2 and 18.5 km per day (Pontzer et al., 2015), while Tsimane forager-horticulturalists engage in relatively low levels of vigorous physical activity (Gurven et al., 2013). Together, these data highlight that ancestral human mobility was flexible and context-dependent, rather than characterised by uniformly high daily movement demands. As such, there is considerable variation in hunter-gatherer mobility we do not suggest that such extreme distances were routinely covered by ancestral groups. Nevertheless, certain behaviours and life history events likely imposed substantial energetic challenges, including but not limited to long-distance travel, persistence hunting and periods of migration. One well-documented example is the colonisation of the Pacific Islands by boat, which involved long and energetically demanding ocean voyages that parallel contemporary ultra-endurance events. These migrations, which involved crossing open ocean for distances of over 1,000 km, required sustained physical effort over extended periods, often under challenging environmental conditions. Voyagers faced exposure to fluctuating temperatures and limited access to resources, creating the potential for substantial negative energy balance (Houghton, 2009; Montenegro et al., 2023).

Thus, while ultra-endurance competitions are not representative of typical ancestral activity patterns, they can provide valuable insight into the physiological demands of rare but evolutionarily significant episodes. Moreover, the utility of studying ultra-endurance events lies in their capacity to induce energetic stress, offering a valuable opportunity to test the core predictions of life history theory (Longman et al., 2020). Specifically, we hypothesised that:

**(H_1_)** Ultra-endurance activity would induce energetic stress, indicated by decreases in body mass and increases in salivary cortisol from pre- to post-race.

**(H_2_)** Trade-offs between multiple key life history functions would be apparent, with defence being prioritised at the expense of storage, maintenance and reproduction, reflecting the greater immediate survival value afforded by defence. Evidence for this will be inferred from side-by-side examination of pre- to post-race changes in biomarkers, with relative increases or conservation of biomarkers of immune defence alongside decreases in biomarkers indexing storage, maintenance and reproductive effort.

## 2. Methods

Male and female athletes were recruited from four multiday ultramarathons and an ocean rowing competition (Table 2). The study was approved by the University of Cambridge Human Biology Research Ethics Committee and written informed consent was obtained from all participants.

**Table 2.** Overview of the 4 ultramarathons and Atlantic Ocean row

Race	Rovaniemi 150 Finland	Jungle Ultra Peru	Al Andalus Ultimate Trail Spain	Everest Trail Race Nepal	World’s Toughest Row Atlantic
Location	Rovaniemi, Finland	Manu National Park, Peru	Granada Province, Spain	Solukhumbu District, Nepal	La Gomera, Spain to Antigua
Climate (temperature & humidity)	Cold (−11–2°C, 90%)	Hot & humid (26–34°C, 77%)	Hot and dry (35–45°C, 35%)	Cool days (5–15°C) & cold nights (−10°C), 50%	Warm days (>25°C) and cool nights (<15°C)
Distance	66/150 km	250 km	230 km	153 km	4,800 km
Mean cumulative race time (days:hours:minutes) (SD)	0:20:15 (0:12:00)	1:15:21 (0:10:07)	1:04:52 (0:8:03)	1:16:07 (0:09:51)	44:03:05 (10:22:44)

Anthropometric measurements, saliva and serum samples were collected before and after the competition. Pre-race measurements were performed 1 or 2 days before the race started, between 10:00 and 16:00. Post-race measurements in Finland, Spain and Antigua were taken 60 minutes after race completion and were not standardised by time. Post-race measurements from Peru and Nepal were taken the day following race completion (due to the remote and inaccessible nature of the finish location) at the same time of day as the pre-race measurements. Inclusion criteria were completion of at least 80% of the race distance and absence of self-reported health conditions or medication use known to influence the measured physiological outcomes. Exclusion criteria included chronic medical conditions, acute illness, or use of medications or substances affecting endocrine, immune or metabolic function. One athlete who self-reported anabolic steroid use via questionnaire was excluded from all analyses.

Stature was measured to the nearest 0.1 cm using a Leicester Stadiometer and body mass to the nearest 0.1 kg using Seca 877 Flat Scales (Seca, Hamburg, Germany) (Longman et al., 2019, 2021). Saliva samples were collected in multiple aliquots using the Salimetrics Saliva Collection Aid (#5016.04). Subjects refrained from eating, drinking, chewing gum, or brushing teeth in the 30 minutes preceding saliva collection (Longman et al., 2017). Venous blood was drawn from the median cubital veins (Chandanathil et al., 2025). See Supplementary Information 1 for further details of the assays.

Body composition was assessed using bioelectric impedance analysis (BIA; BodyStat Quadscan4000). It is appreciated that *in vivo* methods cannot directly measure body composition, but instead provide estimates based on other physiological parameters. BIA was selected for its high precision, simplicity, speed and suitability for monitoring short-term changes in individuals (Achamrah et al., 2018; Wells & Fewtrell, 2006). To estimate FMI from impedance data, we used a regression-based residual approach. Whole body impedance was obtained from the BIA device, and a simple index of 1/impedance was calculated by taking the reciprocal of the measured impedance value. Using pre-race data, body mass index (BMI) was regressed onto 1/impedance and sex to generate predicted BMI values that reflect fat-free mass. FMI was then calculated as the standardised residual from this regression (observed BMI minus predicted BMI, divided by the standard error of the estimate), providing an index of relative fat. This approach avoids the limitations of estimating total body water from impedance and yields a proxy for fat mass adjusted for height, lean mass and sex (Wells et al., 2007). The same regression parameters were applied to post-race data to derive post-race FMI values, allowing within-individual changes in relative fat mass to be assessed over the competition period.

### 2.1. Statistics

Biomarkers are interpreted in terms of within-individual pre-post change rather than relative to sex-specific clinical reference ranges, given the study’s focus on within-individual changes in energetic allocation under acute physiological stress.

A separate linear mixed effects (LME) model was conducted with each log-transformed biomarker as dependent variable. The first set of models estimated the change in the variable from pre- to post-race for the full athlete cohort and included fixed effects for time (pre- vs post-race), age and pre-race BMI, with a random intercept to account for within-subject clustering of repeated measures. Age and pre-race BMI were included as covariates because both can influence baseline biomarker values and the physiological response to energetic stress. To estimate the effect of sex (male vs female), race type (running vs rowing) and self-reported menopausal status in female athletes (pre- vs post-menopausal), a second set of models was conducted for each biomarker including fixed effects for time (pre-race vs post-race), sex (male vs female), sport (runner vs rower), age, pre-race BMI and menopause status, as well as interaction terms were included for time*sex, time*sport and time*menopause status. Random intercepts were included to account for within-subject clustering of repeated measurements. This approach allows the evaluation of each biomarker independently while accounting for repeated measures and potential variation in response due to participant characteristics. All analyses were performed using SPSS v27, and significance set at <0.05.

## 3. Results

Descriptive sample characteristics are presented in Table 3.

**Table 3.** Participant descriptive characteristics, split by event

Variable	Sex	All athletes mean (SD) *n* = 40 f, 107 m	All runners mean (SD) *n* = 33 f, 78 m	Finland mean (SD) *n* = 2 f, 11 m	Peru mean (SD) *n* = 6 f, 13 m	Spain mean (SD) *n* = 20 f, 38 m	Nepal mean (SD) *n* = 5 f, 16 m	Rowers mean (SD) *n* = 7 f, 29 m
Age (years)	F	42.9 (12.0) Range: 19–67	44.2 (10.2)	53.5 (6.4)	33.5 (9.3)	47.8 (9.1)	38.8 (3.1)	36.9 (18.0)
	M	40.7 (12.1) Range: 22–64	44.7 (10.8)	33.6 (7.2)	41.5 (7.5)	49.2 (9.7)	44.4 (11.4)	30.0 (8.8)
Height (cm)	F	165.6 (5.7)	165.7 (5.8)	163.5 (12.7)	162.7 (6.5)	166.1 (5.2)	168.5 (4.3)	164.9 (6.0)
	M	179.8 (6.1)	179.2 (6.1)	180.8 (3.9)	178.9 (3.8)	180.3 (6.8)	175.7 (5.9)	181.3 (6.0)
Body mass (kg)	F	63.1 (7.2)	62.8 (7.4)	65.4 (13.8)	60.8 (4.5)	61.7 (7.3)	68.5 (7.7)	64.5 (6.5)
	M	82.9 (10.9)	79.4 (8.2)	81.2 (5.6)	81.2 (7.8)	78.6 (8.8)	78.6 (8.9)	92.4 (11.8)
BMI (kg/m^2^)	F	23.0 (2.2)	22.8 (2.1)	24.3 (1.4)	22.9 (0.9)	22.2 (2.3)	24.1 (2.0)	23.8 (2.6)
	M	25.6 (2.7)	24.7 (2.1)	24.8 (1.3)	25.3 (2.0)	24.1 (2.1)	25.5 (2.6)	28.0 (2.7)

Age range: 19–67 (male) and 22–64 (female)

**Figure 1.**
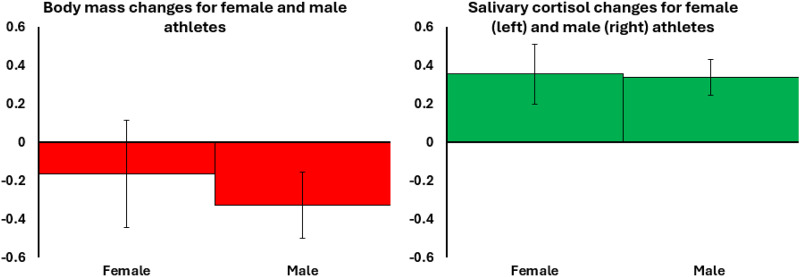
Bar chart illustrating decreases in log-transformed body mass(left) and increases in log-transformed salivary cortisol (right) for female and male athletes (runners and rowers combined, female *n* = 40, male *n* = 107). The *y*-axis shows the model coefficient (B), indicating the magnitude and direction of change. Error bars represent a 95% confidence interval of the B estimate. Coloured bars (green and red) indicate statistically significant changes (increases and decreases).

**Figure 2.**
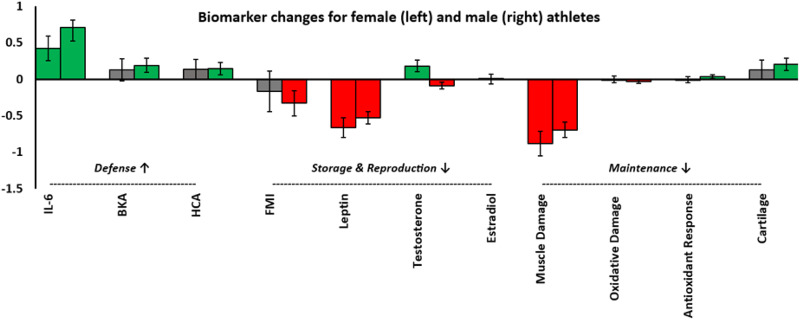
Bar chart showing pre- to post-race changes in log-transformed biomarkers. The bars represent model coefficients (B) derived from linear mixed effects models controlling for age and pre-race BMI. Error bars indicate 95% confidence intervals of the B estimate. Coloured bars (green and red) indicate statistically significant changes (increases and decreases). The chart highlights.

Pre-race and post-race values for all outcome variables are provided in Table 4 and illustrated in Figs. 1 and 2. Supplementary Information 2–5 provides pre- to post-race values for all outcome variables, split by event type (running or rowing), sex, and race location. Supplementary Information 6 provides graphs illustrating the pre- to post-race changes in each biomarker for each race.

**Table 4.** Athletes’ pre- and post-race data, split by sex

	Female athletes			Male athletes		
Variable (*n* = female/male)	Pre-race mean (SD)	Post-race mean (SD)	Mean difference pre-post-race (SD)	Pre-race mean (SD)	Post-race mean (SD)	Mean difference pre-post-race
Body mass (kg) (*n* = 40/107)	63.1 (7.2)	60.3 (6.7)	−2.8 (3.1)	82.9 (10.9)	78.4 (8.6)	−4.5 (4.9)
Cortisol (μg/dL) (*n* = 31/72)	0.1 (0.1)	0.2 (0.3)	0.2 (0.4)	0.1 (0.1)	0.4 (0.4)	0.2 (0.4)
IL-6 (pg/mL) (*n* = 35/93)	2.8 (1.8)	8.5 (6.9)	5.7 (6.7)	1.5 (1.5)	7.7 (8.8)	6.2 (8.6)
BKA (%) (*n* = 39/103)	0.5 (0.3)	0.6 (0.4)	0.0 (0.3)	0.4 (0.4)	0.5 (0.3)	0.2 (0.3)
HCA (CH50) (*n* = 37/103)	0.1 (0.1)	0.0 (0.0)	0.0 (0.1)	0.1 (0.1)	0.0 (0.1)	0.0 (0.2)
Fat mass index (*n* = 37/104)	0.0 (1.0)	−0.5 (0.9)	−0.5 (0.3)	0.0 (1.0)	−0.4 (1.0)	−0.4 (0.4)
Leptin (ng/mL) (*n* = 39/104)	6.2 (4.3)	1.9 (2.2)	−4.3 (3.4)	5.0 (10.6)	2.9 (9.3)	−2.0 (4.2)
Testosterone (pg/mL) (*n* = 38/102)	1.3 (1.0)	2.1 (1.6)	0.8 (1.6)	91.5 (47.5)	76.6 (41.1)	−15.0 (39.7)
Oestradiol (pg/mL) (*n* = 37)	48.2 (36.5)	48.3 (38.4)	0.1 (32.2)	–	–	–
Myoglobin (ng/mL) (*n* = 38/102)	109.8 (52.6)	1,662.6 (2,173.6)	1,552.8 (2,159.3)	222.2 (231.1)	1,552.1 (1,790.4)	1,329.9 (1,814.2)
MDA (pmol/mL) (*n* = 39/104)	202.6 (127.5)	194.2 (117.5)	−8.4 (72.1)	127.9 (83.9)	136.0 (83.1)	8.1 (39.8)
TAC (mM) (*n* = 39/105)	0.9 (0.4)	0.8 (0.3)	0.0 (0.3)	0.7 (0.3)	0.8 (0.3)	0.1 (0.2)
COMP (pg/mL) (*n* = 38/99)	6,710.0 (6,108.9)	4,818.5 (3,839.9)	−1,891.6 (3,582.7)	2,816.2 (2,505.8)	1,674.1 (1,148.9)	−1,142.1 (2,671.4)

- An increase in energetic investment towards biomarkers of defence, as indicated by increase in interleukin-6 (IL-6), bacteria killing assay (BKA) and haemolytic complement activity (the haemolytic complement assay revealed a pre- to post-race decrease in absorbance, representative of an increase in activity. This is shown as an increase here)/
- A decrease in investment towards biomarkers of storage, as shown by decreases in fat mass index (FMI) and leptin.
- A general decrease in investment towards biomarkers of reproduction, with decreased FMI and leptin in females and decreased testosterone in males.
- A general decrease in biomarkers of maintenance, evidenced by increases in biomarkers of muscle and oxidative damage (shown as a decrease in Fig. 2), alongside an increase in Total Antioxidant Capacity (TAC) and Cartilage Oligomeric Protein Matrix (COMP).

**H_1_: Ultra-endurance activity induces energetic stress.**

Event participation was associated with both energetic costs and physiological stress, reflected in reductions in FMI and body mass, alongside an increase in cortisol (Table 4; Fig. 1). These effects were consistent across sex, sport type, and menopausal status, although the magnitude and significance varied between subgroups.

**H_2_: Trade-offs between multiple key life history functions would be apparent, with defence being prioritised at the expense of storage, maintenance and reproduction.**

1. **Defence**

Biomarkers indicative of investment in defence increased in male athletes and were generally maintained in female athletes.

Event participation was associated with increased investment in defence, reflected in significant elevations of IL-6 and BKA, alongside enhanced HCA (Table 5; Fig. 2). These effects were generally stronger in male athletes, although increases were evident across sex, sport type and menopausal status, with varying magnitudes and statistical significance.

1. **Storage**

**Table 5.** Regression coefficients (B), 95% confidence intervals and *p*-values for changes in biomarkers from pre- to post-race, controlling for age and pre-race BMI

Variable	Overall	Male	Female	Runners	Rowers	Pre-menopause	Post-menopause
Fat mass index	−0.29 [−0.44, −0.01], *p* < .001	−0.33 [−0.50, −0.16], *p* < .001	−0.16 [−0.44, 0.12], *p* = .249	−0.21 [−0.37, −0.06], *p* = .007	−0.70 [−1.01, −0.38], *p* < .001	−0.26 [−0.43, −0.09], *p* = .004	−0.15 [−0.36, 0.06], *p* = .153
Body mass	−0.02 [−0.03, −0.02], *p*< .001	−0.02 [−0.03, −0.02], *p* < .001	−0.02 [−0.03, −0.01], *p* < .001	−0.01 [−0.01, −0.01], *p* < .001	−0.06 [−0.06, −0.05], *p* < .001	−0.02 [−0.02, −0.01], *p* < .001	−0.03 [−0.04, −0.02], *p* < .001
Cortisol	0.34 [0.26, 0.42], *p* < .001	0.34 [0.25, 0.43], *p* < .001	0.36 [0.20, 0.51], *p* < .001	0.37 [0.28, 0.47], *p* < .001	0.24 [0.08, 0.40], *p* = .003	0.24 [0.05, 0.43], *p* = .013	0.62 [0.32, 0.91], *p* < .001
IL-6	0.63 [0.54, 0.72], *p* < .001	0.71 [0.61, 0.82], *p* < .001	0.42 [0.25, 0.60], *p* < .001	0.68 [0.58, 0.78], *p* < .001	0.45 [0.26, 0.64], *p* < .001	0.37 [0.24, 0.50], *p* < .001	0.57 [0.36, 0.78], *p* < .001
BKA	0.17 [0.09, 0.25], *p* < .001	0.19 [0.09, 0.29], *p* < .001	0.13 [−0.02, 0.28], *p* = .095	0.23 [0.14, 0.33], *p* < .001	0.01 [−0.15, 0.17], *p* = .158	0.18 [−0.02, 0.37], *p* = .073	−0.00 [−0.32, 0.31], *p* = .978
HCA*	−0.14 [−0.21, −0.07], *p* < .001	−0.14 [−0.23, −0.06], *p* < .001	−0.14 [−0.27, 0.00], *p* = .053	−0.17 [−0.25, −0.09], *p* < .001	−0.05 [−0.20, 0.10], *p* = .519	−0.17 [−0.34, 0.01], *p* = .057	−0.04 [−0.32, 0.24], *p* = .636
Leptin	−0.56 [−0.63, −0.49], *p* < .001	−0.53 [−0.61, −0.44], *p* < .001	−0.66 [−0.77, −0.31], *p* < .001	−0.50 [−0.58, −0.43], *p* < .001	−0.76 [−0.90, −0.61], *p* < .001	−0.61 [−0.77, −0.45], *p* < .001	−0.80 [−1.06, −0.54], *p* < .001
Testosterone	−0.01 [−0.06, 0.03], *p* = .587	−0.09 [−0.13, −0.04], *p* < .001	0.18 [0.10, 0.26], *p* < .001	−0.05 [−0.10, 0.01], *p* = .094	0.08 [−0.01, 0.18], *p* = .072	0.14 [0.01, 0.28], *p* = .039	0.29 [0.07, 0.52], *p* = .012
Oestradiol (females only)	–	–	0.09 [−0.06, 0.07], *p* = .932	0.00 [−0.07, 0.08], *p* = .920	−0.00 [−0.16, 0.16], *p* = .987	0.02 [−0.06, 0.10], *p* = .666	−0.04 [−0.17, 0.09], *p* = .584
Myoglobin	0.74 [0.65, 0.83], *p* < .001	0.69 [0.59, 0.80], *p* < .001	0.88 [0.71, 1.05], *p* < .001	0.86 [0.77, 0.95], *p* < .001	0.37 [0.20, 0.54], *p* < .001	0.86 [0.65, 1.08], *p* < .001	0.93 [0.59, 1.26], *p* < .001
Malondialdehyde	0.02 [−0.00, 0.05], *p* = .072	0.03 [0.00, 0.06], *p* = .037	0.00 [−0.05, 0.05], *p* = .969	0.02 [−0.01, 0.14], *p* = .103	0.02 [−0.03, 0.07], *p* = .446	0.01 [−0.06, 0.08], *p* = .828	−0.02 [−0.13, 0.09], *p* = .773
Total Antioxidant Capacity	0.03 [0.00, 0.05], *p* = .021	0.04 [0.01, 0.06], *p* = .004	−0.01 [−0.05, 0.04], *p* = .816	0.04 [0.02, 0.06], *p* = .001	−0.02 [−0.07, 0.02], *p* = .306	−0.03 [−0.07, 0.02], *p* = .209	0.05 [−0.02, 0.12], *p* = .128
Cartilage Oligomeric Matrix Protein	−0.19 [−0.26, −0.11], *p* < .001	−0.21 [−0.29, −0.12], *p* < .001	−0.13 [−0.27, 0.01], *p* = .056	−0.25 [−0.33, −0.17], *p* < .001	−0.01 [−0.14, 0.15], *p* = .941	−0.17 [−0.26, −0.07], *p* = .001	−0.04 [−0.19, 0.11], *p* = .113

*Note*:

* Negative values for HCA represent increased haemolytic complement activity.

Biomarkers associated with energy storage declined following race participation, consistent with mobilisation of reserves under energetic stress (Table 5; Fig. 2). Both FMI (described above) and leptin decreased significantly across the sample, with comparable declines across subgroups.

1. **Reproduction**

Markers of reproductive investment decreased in males and broadly declined in females (Table 5, Fig. 2). Testosterone decreased in male athletes, while female testosterone increased and oestradiol remained unchanged. FMI and leptin considered markers of female reproductive investment, both decreased following race participation. The reduction in FMI for females (*B* = –0.163) was smaller than in males (*B* = –0.328), suggesting a sex difference in fat mass preservation, with females showing greater protection against fat loss under energetic stress.

1. **Maintenance**

Biomarkers associated with investment in maintenance generally decreased following race completion.

Markers of maintenance indicated greater cellular damage and compensatory responses following race participation (Table 5; Fig. 2). Myoglobin increased across all subgroups, consistent with muscle damage. MDA, which tended to increase in males but not females and varied between races (increasing after the Peru and Spain races, decreasing after the Spain race and remaining unchanged after the Finland race). TAC increased in males (but not females) and in runners (but not rowers). COMP, a marker of cartilage turnover, decreased overall, although this was not observed in rowers.

## 4. Discussion

This study provides evidence suggestive of the existence of trade-offs between biomarkers of multiple life history functions in humans. Specifically, biomarkers of defence were broadly prioritised relative to storage, reproduction and maintenance.

The first hypothesis was supported by the pronounced loss of body mass experienced by athletes participating in the ultra-endurance events. While a proportion of this mass loss likely reflects acute dehydration, the magnitude indicates substantial energetic stress. This energetic stress was accompanied by an increase in cortisol, indicative of increased activation of the hypothalamic-adrenal-pituitary axis stress response pathway. As a gateway hormone regulating metabolism, cortisol acts as a central regulator of multiple physiological systems (Crespi et al., 2013; Oakley & Cidlowski, 2013) and may have contributed to the observed trade-offs. Pre-race cortisol was greater in rowers than runners, potentially reflecting greater anticipatory anxiety before undertaking the longer and riskier Atlantic rowing event.

With some variation across the five races and between male and female athletes, biomarkers of defence were generally enhanced or preserved, whereas biomarkers of investment in storage, reproduction and maintenance declined, supporting the second hypothesis. Considering defence, IL-6 levels increased in both sexes. In male athletes, immune function biomarkers (BKA and HCA) increased significantly, whereas in female athletes these measures showed a non-significant trend towards increasing. In contrast, biomarkers of investment in energy storage decreased in both sexes, as indicated by reduction in leptin – which signals energy status to the brain to inform energy allocation – and decreased FMI (a non-significant trend for females). Similarly, reproductive investment broadly declined: male athletes exhibited reduced testosterone, while female athletes showed decreases in FMI and leptin, no change in oestradiol and an increase in testosterone. The latter may reflect a typical phenotypic response to competition (Bateup et al., 2002; Longman, Surbey, et al., 2018) rather than increased investment in reproductive effort.

Biomarkers related to maintenance reflected substantial structural and cellular damage following race participation, consistent with the extreme mechanical and metabolic demands of prolonged ultra-endurance activity. An unexpected decrease in the cartilage damage biomarker (COMP) was observed in runners but not rowers; this may reflect depletion of COMP. Antioxidant protection (TAC) increased in male athletes but remained unchanged in females. Sex-specific patterns in maintenance-related biomarkers may also partly reflect the modulatory effects of oestrogens, which exhibit antioxidant and anti-inflammatory properties. Experimental evidence suggests that oestradiol can attenuate exercise-induced muscle damage and strength loss (Minahan et al., 2015), potentially influencing the magnitude or expression of maintenance costs under energetic stress. These patterns could reflect an alternative to a simple decrease in maintenance: over the timescale of this study (several days to weeks of energetic deficit), athletes’ bodies may have attempted to maintain tissue integrity and prevent damage, but the energetic demands of ultra-endurance activity exceeded available resources. In other words, maintenance processes may have been engaged, but the resources available were insufficient to fully prevent muscle and oxidative damage. The extent to which compensatory mechanisms might operate over shorter or longer timescales, or whether maintenance could be preserved under less extreme stress, remains to be determined.

Taken together, these results suggest that, despite some sex-specific differences in individual biomarkers, both male and female athletes exhibited broadly similar patterns of resource allocation under extreme energetic stress. Reproductive and storage biomarkers decreased in both sexes, indicating suppression of these functions, while defence biomarkers generally increased – significantly for all three measures in males and in one measure for females, with trends toward increasing in the other two (potentially reflecting the smaller female sample size). Overall, these findings support the existence of energy allocation trade-offs in both sexes, and future work could use a life history framework to further elucidate the hierarchy of functional preservation and how it may vary both intra- and inter-individually with factors such as population, age, sex, and body composition.

Sex differences in body composition may also contribute to variation in maintenance-related biomarkers. Males typically possess greater absolute and relative fat-free mass, and lower fat mass, which may influence both the magnitude of exercise-induced muscle damage and the energetic resources available for tissue repair under conditions of energetic stress. While body mass and FMI declined in both sexes, further work incorporating detailed changes in fat-free mass may help refine interpretations of sex-specific damage and recovery trajectories.

Both runners and rowers exhibited broadly similar patterns of biomarker changes, and the pattern was consistent across the four running races. Both sports showed substantial energetic stress, as indicated by reductions in body mass, FMI and increases in cortisol. Considering trade-offs, both groups displayed a general prioritisation of defence: runners exhibited significant increases in all three biomarkers, whereas rowers showed a significant increase in one biomarker and a non-significant trend in another. Biomarkers of energy storage decreased in both groups, while reproductive and maintenance biomarkers generally declined. Although running and rowing impose different physiological demands – for example, running is weight-bearing and imposes significant ground reaction forces, while rowing does not – the consistent pattern of biomarker changes across both sports suggests that energetic stress was the primary driver of these responses. Some sport-specific nuances were observed: rowers lost more body mass, consistent with the longer race duration, whereas male runners exhibited larger increases in IL-6, BKA, myoglobin, and COMP, and running appeared to more strongly suppress testosterone in both sexes. Likewise, the magnitude and nature of the changes observed exceed those typically induced by moderate exercise, indicating that the effects were driven primarily by the energetic stress rather than by sport-specific mechanics (Olive & Miller, 2001; Parker et al., 2014; Scalco et al., 2016). Overall, despite these minor differences, the energetic stresses imposed by both sports appear to elicit consistent trade-offs in energy allocation, with defence generally prioritised at the expense of storage, reproduction and maintenance.

There is significant interdisciplinary interest in understanding patterns of energy allocation during conditions of energetic stress. Our evolutionary history, marked by repeated cycles of dispersal (Wells & Stock, 2007), likely exposed migrating humans to a variety of energetic challenges. For instance, elevated immune activation – an energetically costly process (Segerstrom, 2007) – in high-pathogen, low-resource environments in our ancestral past would have imposed significant energetic stress (Urlacher et al., 2018), leading to powerful selective pressures for energetic efficiency. Research seeking to understand the competitive allocation of resources thus explores physiological plasticity and the adaptive strategies that enabled humans to persist across ecologically diverse habitats (Stock et al., 2023; Wells & Stock, 2007).

These insights also have contemporary relevance. Energetic stress remains widespread, whether from food insecurity – affecting nearly one in three people globally (∼2.37 billion) (UNICEF, 2021) – or increased energetic demands associated with infection (Muehlenbein et al., 2010), pregnancy or lactation (Ellison, 2003). While patterns of energy allocation underpin multiple functional relationships, interpretation of results from endurance exercise as a model of energetic stress more broadly requires caution. In context of food insecurity, for example, trade-offs may not mirror those observed in athletes: reproduction is often maintained rather than curtailed, with implications for child malnutrition (Vitzthum, 2009). Recognising the diversity of adaptive responses is critical to health and medical outcomes. Increased knowledge of the scope of human plasticity and the adaptive response to energetic stress outside the context of overt disease can inform numerous areas of public health, advancing understanding of body weight regulation, physical activity, diet and health. This perspective is central to the emerging field of evolutionary public health, which applies knowledge derived from life history theory trade-offs to improve the efficacy of public health interventions (Muehlenbein, 2006, 2010; Muehlenbein et al., 2005; Muehlenbein & Bribiescas, 2005; Wells et al., 2017). Similarly, athletes across a range of athletic disciplines regularly undergo planned periods of energetic stress to reduce body fat or enhance metabolic efficiency (Shirley et al., 2022; Stellingwerff et al., 2019). As prolonged exposure to energy deficit increases injury risk, compromises bone health and disrupts reproductive function (Areta et al., 2020; Mountjoy et al., 2018; Nattiv et al., 2007), exercise physiologists seek to better characterise patterns of energy allocation to optimise both performance and health (Longman et al., 2023).

The strengths of this study include the consideration of all four life history functions (facilitated by the broad suite of biomarkers analysed), the large sample size and the broadly consistent results between the sexes and across ultra-endurance competitions varying both by climate, discipline (including an age difference between runners and rowers) and duration. Nonetheless, caution is warranted in generalising beyond this sample of highly trained athletes, whose physical conditioning may buffer against the physiological consequences of energetic stress during prolonged and strenuous physical exercise. However, the universality of general life history theory predictions suggest that broadly similar patterns may occur in different populations, given the evolutionary (Carrier, 1984; Lieberman et al., 2006; Longman et al., 2015; Ocobock & Lacy, 2023) and cross-cultural ecological relevance of endurance running (Liebenberg, 2006; Pennington, 1963). We also acknowledge that ancestral populations did not perform physical activity to the same degree of intensity and duration as the athletes in this study. Additionally, some biomarkers reported serve multiple physiological roles, complicating their assignment to specific life history functions (e.g., testosterone in females). Sex-specific considerations also warrant acknowledgment. While analyses distinguished between pre- and post-menopausal females, hormonal variation across the menstrual cycle in premenopausal female athletes was not standardised and hormonal contraceptive use was not controlled for. Given the logistical challenges of field-based research at ultra-endurance competitions, standardising testing to specific cycle phases was not feasible. This variability may have increased within-group variance and reduced statistical power for detecting female-specific effects, rather than systematically biasing results. Finally, although this study includes a larger number of female ultra-endurance athletes than most comparable work, the female sample remained smaller than the male sample, which may further limit power to detect sex-specific effects. Methodological variation must also be noted: post-race samples in Finland and Spain were taken within 60-minutes of finishing, whereas those in Peru and Nepal were collected 24 hours after finishing due to the inaccessibility of the remote finish locations. Supplementary analyses, however, confirmed that this did not alter patterns for any key biomarker (Supplementary Information 3 and 4). While post-race samples were not standardised by time of day, raising potential circadian influences, these appear unlikely to explain the main findings.

In conclusion, this study provides experimental evidence for the existence of trade-offs between biomarkers of multiple life history functions. Specifically, biomarkers of defence were broadly prioritised relative to storage, reproduction and maintenance. This quasi-experimental approach, incorporating a wide biomarker panel, a large sample size and observing consistency across sex, climate, and event duration, lends robustness to the findings. Future work may seek to further clarify the hierarchy of functional preservation and how it varies both intra- and inter-individually with factors such as population, age, sex, and body composition.

## Supporting information

10.1017/ehs.2026.10040.sm001 Longman et al. supplementary material Longman et al. supplementary material

## Supplementary material

The supplementary material for this article can be found at https://doi.org/10.1017/ehs.2026.10040.

## Research transparency and reproducibility interest

Data will be made available via Loughborough University’s online Research Repository.
