# Supplementary material for: Experimental evidence of life history trade-offs during ultra-endurance physical activity
Source: Evol Hum Sci. 2026 Mar 6;8:e14. doi: 10.1017/ehs.2026.10040 (PMC13168829; doi:10.1017/ehs.2026.10040)
Supplement: Longman et al. supplementary material [file S2513843X26100401sup001.docx]

**Supplementary Information 1. Assay details.**

Serum samples were frozen at -20°C at field sites and shipped to the University of Cambridge where they were stored at -80°C before being shipped to Baylor University on dry ice. Upon arrival, all samples were immediately placed into -80°C freezers; no samples thawed in transit. All assays were performed between January and May 2020. The assay kits (commercially available where indicated), number freeze/thaw cycles of samples needed to perform each assay and intra- and inter-assay coefficients of variation are displayed below in Table S7. All samples were run in duplicate (triplicate for the Bacteria Killing Assay), and all ELISA tests were conducted with the same person’s samples as well as the same race/event run on the same microplate. The Bacteria Killing assay is a functional measure of serum ability to kill bacteria. The assay is performed by mixing serum and known number of bacteria (*E.coli* ATCC #8739) in a 1:20 ratio and spreading it on trypticase soy agar plates in triplicates. After overnight incubation, colony forming units are counted using the Protos 3 system. Percent killing is calculated by comparing how many bacteria colonies grow after exposure to the diluted sample relative to a positive growth control.

| **Analyte** | **Assay** | **Freeze-thaw cycles** | **Inter-assay CV (%)** | **Intra-assay CV (%)** |
| --- | --- | --- | --- | --- |
| TAC | CAYMAN 709001 | 1 | 5.1 | 9.0 |
| COMP | ABCAM ab213764 | 1 | 8.40 | 9.1 |
| IL-6 | ABCAM ab46042 | 1 | 13.4 | 19.0 |
| Malondialdehyde | ABCAM ab238537 | 1 | 5.9 | 6.5 |
| Leptin | ALPCO 11-LEPHU-E01 | 1 | 8.0 | 5.1 |
| Cortisol | ABCAM ab108665 | 2 | 11.1 | 7.0 |
| Myoglobin | ABCAM ab108652 | 2 | 3.4 | 7.3 |
| BKA | In-house assay | 2 | 14.6 | N/A |
| HCA | In-house assay | 2 | 2.3 | 3.6 |
| Estradiol | ALPCO 11-ESTHU-E01 | 2 | 11.4 | 9.9 |
| Testosterone | ALPCO 11-FTEHU-E01 | 3 | 7.8 | 2.5 |

**Table S6.** The assay kits (commercially available where indicated), number freeze/thaw cycles of samples needed to perform each assay and intra- and inter-assay coefficients of variation.

**Supplementary Information 2. Runners’ pre- and post-race data, split by sex.**

|  | **Female runners** |  | **Male runners** |  |
| --- | --- | --- | --- | --- |
| **Variable**  **(*n* = female/male)** | **Pre-race Mean (SD)** | **Post-race Mean (SD)** | **Pre-race Mean (SD)** | **Post-race Mean (SD)** |
| Body mass (kg)  (*n*=33/78) | 62.8 (7.4) | 61.0 (7.1) | 79.4 (8.2) | 77.4 (8.2) |
| Cortisol (μg/dL)  (*n*=31/72) | 0.1 (0.0) | 0.2 (0.3) | 0.1 (0.1) | 0.4 (0.4) |
| IL-6 (pg/mL)  (*n*=29/71) | 2.6 (1.4) | 9.1 (7.2) | 1.6 (1.6) | 9.2 (9.5) |
| BKA (%)  (*n*=32/77) | 0.5 (0.3) | 0.5 (0.4) | 0.3 (0.3) | 0.5 (0.3) |
| HCA (CH50)  (*n*=30/78) | 0.1 (0.1) | 0.0 (0.0) | 0.1 (0.2) | 0.0 (0.1) |
| Fat mass index  (n=33) | 0.0 (1.0) | -0.5 (0.9) | 0.0 (1.0) | -0.4 (1.0) |
| Leptin (ng/mL)  (*n*=32/78) | 6.4 (4.7) | 1.9 (2.3) | 4.2 (10.4) | 2.8 (9.1) |
| Testosterone (pg/mL)  (*n*=31/74) | 1.3 (1.0) | 1.8 (1.2) | 99.7 (50.4) | 79.3 (45.1) |
| Oestradiol (pg/mL)  (*n*=30) | 49.0 (35.7) | 49.9 (40.8) | - | - |
| Myoglobin (ng/mL)  (*n*=31/78) | 110.4 (48.6) | 1,944.2 (2,314.4) | 183.6 (97.2) | 1,886.8 (1,971.8) |
| MDA (pmol/mL)  (*n*=32/77) | 212.2 (135.8) | 207.9 (123.5) | 138.7 (90.9) | 144.4 (86.8) |
| TAC (mM)  (*n*=32/78) | 0.9 (0.4) | 0.9 (0.3) | 0.7 (0.2) | 0.8 (0.3) |
| COMP (pg/mL)  (*n*=31/76) | 7,643.3 (6,375.6) | 5,315.4 (4,005.5) | 3,090.1 (2,762.2) | 1,396.0 (977.6) |

**Table S2.** Pre- and post-race data for runners, split by sex.

**Supplementary Information 3. Female runners’ pre- and post-race data, split by race.**

|  | **Finland (n=2)** |  | **Peru (n=6)** |  | **Spain (n=20)** |  | **Nepal (n=5)** |  | |
| --- | --- | --- | --- | --- | --- | --- | --- | --- | --- |
| **Variable** | **Pre-race Mean (SD)** | **Post-race Mean (SD)** | **Pre-race Mean (SD)** | **Post-race Mean (SD)** | **Pre-race Mean (SD)** | **Post-race Mean (SD)** | **Pre-race Mean (SD)** | **Post-race Mean (SD)** | |
| Body mass (kg) | 65.4 (13.8) | 62.7 (13.4) | 60.8 (4.5) | 59.0 (4.8) | 61.7 (7.3) | 59.8 (6.5) | 68.5 (7.7) | 67.2 (8.0) | |
| Cortisol (μg/dL) | 0.1 (0.0) | 0.4 (0.2) | 0.1 (0.0) | 0.0 (0.0) | 0.1** (0.1) | 0.3** (0.4) | 0.1 (0.1) | 0.1 (0.1) | |
| IL-6 (pg/mL) | 2.1* (-) | 13.6* (-) | 4.4* (1.2) | 5.5* (2.5) | 2.1** (1.1) | 10.7** (8.1) | 2.3 (1.3) | 5.8 (5.2) | |
| BKA (%) | 0.5 (0.7) | 0.8 (0.2) | 0.7 (0.3) | 0.7 (0.4) | 0.5* (0.3) | 0.5* (0.3) | 0.3 (0.2) | 0.2 (0.4) | |
| HCA (CH50) | 0.0 (0.0) | 0.0 (0.1) | 0.0* (0.0) | 0.0* (0.0) | 0.0** (0.0) | 0.0** (0.0) | 0.3 (0.2) | 0.0 (0.0) | |
| Fat mass index | 0.3 (1.0) | 0.2 (1.1) | 0.3 (0.8) | -0.4 (0.6) | -0.2 (1.0) | -0.6 (1.0) | 0.3 (1.2) | -0.1 (1.0) | |
| Leptin (ng/mL) | 2.5 (2.7) | 0.2 (0.0) | 9.5 (2.8) | 3.7 (2.4) | 5.7* (5.1) | 1.7* (2.4) | 6.8 (4.4) | 1.0 (0.6) | |
| Testosterone (pg/mL) | 0.5* | 2.5* | 1.6 (1.9) | 1.0 (0.7) | 1.3* (0.7) | 2.3* (1.2) | 1.1 (1.1) | 0.8 (0.8) | |
| Oestradiol (pg/mL) | 75.4* | 97.1* | 58.6* (25.8) | 38.5* (9.4) | 51.3* (40.5) | 56.6* (46.8) | 25.5 (13.3) | 26.3 (21.8) | |
| Myoglobin (ng/mL) | 211.2 (52.8) | 6,782.9 (3,976.7) | 137.1* (39.2) | 292.5* (152.5) | 92.1* (33.8) | 2,328.8* (1,918.5) | 112.9 (52.7) | 198.6 (69.5) | |
| MDA (pmol/mL) | 232.7 (20.6) | 279.5 (109.2) | 162.0* (137.0) | 253.8* (185.9) | 258.9* (136.6) | 209.2* (108.5) | 87.1 (38.7) | 119.0 (58.7) | |
| TAC (mM) | 1.1 (0.5) | 1.0 (0.4) | 1.2 (0.8) | 1.1 (0.3) | 0.8* (0.2) | 0.8* (0.2) | 0.8 (0.1) | 0.8 (0.2) | |
| COMP (pg/mL) | 9,975.8 (10,904.1) | 7,619.5 (4,515.4) | 11,432.5* (1,436.4) | 9,178.8* (981.7) | 4,280.8* (4,065.2) | 3,743.6* (3,863.7) | 15,698.5 (6,574.6) | 6,503.4 (3,504.5) | |
| *Indicates a sample size one fewer than stated at the column heading; **Indicates a sample size two fewer than stated at the column heading. Sample sizes may differ across biomarkers due to practical constraints, including limited sample volume and assay requirements. | | | | | | | | |  |

**Table S3.** Pre- and post-race data for female runners, split by race.

Race logistics required that post-race measurements be taken one hour after completion of the Finland and Spain races (total n=22) and the following day after completion of the Peru and Nepal races (total n=11). Additional analyses were conducted to assess whether the inclusion of the Peru and Nepal samples affected the female pattern of results. Inclusion of the Peru and Nepal samples did not change the overall interpretation of the results. Their inclusion has the following effects:

- Energetic stress: unchanged results for Δbody mass or Δcortisol.
- Defense: unchanged results for ΔIL-6 or ΔBKA or ΔHCA.
- Storage: unchanged results for Δfat mass index or Δleptin.
- Reproduction: unchanged results for Δestradiol or Δtestosterone.
- Maintenance: unchanged results for Δmyoglobin, ΔTAC or ΔCOMP. Finland & Spain athletes exhibited a decrease in MDA, while Peru and Nepal athletes exhibited an increase.

**Supplementary Information 4. Male runner pre- and post-race data, split by race.**

|  | **Finland (n=11)** |  | **Peru (n=13)** |  | **Spain (n=38)** |  | **Nepal (n=16)** |  |
| --- | --- | --- | --- | --- | --- | --- | --- | --- |
| **Variable** | **Pre-race Mean (SD)** | **Post-race Mean (SD)** | **Pre-race Mean (SD)** | **Post-race Mean (SD)** | **Post-race Mean (SD)** | **Post-race Mean (SD)** | **Pre-race Mean (SD)** | **Post-race Mean (SD)** |
| Body mass (kg) | 81.2 (5.6) | 79.1 (5.9) | 81.2 (7.8) | 80.0 (7.6) | 78.6 (8.8) | 75.6 (8.8) | 78.6 (8.9) | 77.5 (8.3) |
| Cortisol (μg/dL) | 0.2***(0.1) | 0.6***(0.5) | 0.1* (0.0) | 0.2* (0.3) | 0.1* (0.1) | 0.5* (0.4) | 0.2* (0.1) | 0.1* (0.1) |
| IL-6 (pg/mL) | 0.8**** (0.7) | 18.1 (11.8) | 2.6 (0.8) | 9.9 (14.0) | 1.4*** (1.9) | 8.7*** (6.7) | 1.5 (1.4) | 6.0 (7.3) |
| BKA (%) | 0.5 (0.4) | 0.6 (0.3) | 0.3 (0.3) | 0.5 (0.3) | 0.3* (0.3) | 0.4 (0.3) | 0.1 (0.2) | 0.4 (0.3) |
| HCA (CH50) | 0.0 (0.0) | 0.0 (0.0) | 0.0 (0.0) | 0.0 (0.0) | 0.0 (0.0) | 0.0 (0.1) | 0.3 (0.3) | 0.0 (0.1) |
| Fat mass index | 0.3 (0.8) | 0.4 (0.8) | 0.3 (1.0) | -0.4 (1.0) | -0.3 (0.9) | -0.6 (0.9) | 0.3 (1.1) | -0.2 (1.0) |
| Leptin (ng/mL) | 2.1 (2.2) | 0.6 (0.8) | 2.4 (3.0) | 1.2 (1.9) | 6.4 (14.5) | 4.9 (12.7) | 2.1 (2.8) | 0.5 (0.6) |
| Testosterone (pg/mL) | 161.3*** (49.4) | 100.3*** (20.6) | 133.5 (21.5) | 107.0 (23.6) | 90.5* (42.1) | 85.9* (48.4) | 62.7 (43.9) | 30.9 (10.0) |
| Myoglobin (ng/mL) | 119.8*** (32.2) | 3,566.3 (2,116.5) | 239.2 (145.0) | 375.3 (237.4) | 171.9 (61.5) | 2,646.7 (1,998.9) | 198.0 (120.7) | 470.3 (320.3) |
| MDA (pmol/mL) | 154.5 (64.1) | 151.0 (72.9) | 124.3 (45.9) | 179.1 (50.3) | 168.9* (110.5) | 152.3* (107.9) | 69.7 (20.6) | 93.4 (24.4) |
| TAC (mM) | 0.7 (0.1) | 0.8 (0.2) | 0.7 (0.2) | 0.8 (0.3) | 0.6 (0.3) | 0.7 (0.3) | 0.9 (0.1) | 1.1 (0.4) |
| COMP (pg/mL) | 3,237.1 (1,507.5) | 2,240.8 (668.6) | 1,905.0 (863.9) | 903.5 (488.9) | 1,798.3*** (1,233.8) | 1,391.9*** (1,101.7) | 7,212.5** (3,358.8) | 1,199.6** (835.0) |
| *Indicates a sample size one fewer than stated at the column heading; **Indicates a sample size two fewer than stated at the column heading; ***Indicates a sample size three fewer than stated at the column heading. Sample sizes may differ across biomarkers due to practical constraints, including limited sample volume and assay requirements. | | | | | | | | |

**Table S4.** Pre- and post-race data for male runners, split by race.

Race logistics required that post-race measurements be taken one hour after completion of the Finland and Spain races (total n=49) and the following day after completion of the Peru and Nepal races (total n=39). Additional analyses were conducted to assess whether the inclusion of the Peru and Nepal samples affected the male pattern of results. Inclusion of the Peru and Nepal samples did not change the overall interpretation of the results. Their inclusion has the following effects:

- Energetic stress: unchanged results for Δbody mass or Δcortisol.
- Defense: unchanged results for ΔIL-6 or ΔBKA. The overall male decrease in HCA appears to have been driven by the Nepal cohort, which was the only race to exhibit a decrease.
- Storage: unchanged results for Δfat mass index or Δleptin.
- Reproduction: unchanged results for Δtestosterone.
- Maintenance: unchanged results for Δmyoglobin or ΔCOMP. Finland & Spain athletes exhibited a decrease in MDA and no change in TAC, while Peru and Nepal athletes exhibited an increase in MDA and an increase in TAC.

**Supplementary Information 5. Rower pre- and post-race data.**

|  | **Female rowers** |  | **Male rowers** |  |
| --- | --- | --- | --- | --- |
| **Variable**  **(*n* = female/male)** | **Pre-race Mean (SD)** | **Post-race Mean (SD)** | **Pre-race Mean (SD)** | **Post-race Mean (SD)** |
| Body mass (kg)  (*n*=7/29) | 64.5 (6.5) | 56.8 (3.4) | 92.4 (11.8) | 81.0 (9.2) |
| Cortisol (μg/dL)  (*n*=29) | 0.1 (0.1) | 0.3 (0.4) | 0.2 (0.1) | 0.3 (0.2) |
| IL-6 (pg/mL)  (*n*=6/22) | 4.2 (3.1) | 6.0 (5.5) | 1.0 (0.9) | 2.7 (2.0) |
| BKA (%)  (*n*=7/26) | 0.7 (0.4) | 0.7 (0.5) | 0.7 (0.3) | 0.7 (0.3) |
| HCA (CH50)  (*n*=7/25) | 0.0 (0.0) | 0.0 (0.0) | 0.0 (0.0) | 0.0 (0.0) |
| Fat mass index  (n=4/26) | 0.0 (1.0) | -1.0 (0.3) | 0.0 (1.0) | -1.7 (1.0) |
| Leptin (ng/mL)  (*n*=7/26) | 5.3 (1.6) | 2.2 (2.1) | 7.1 (11.0) | 3.3 (9.9) |
| Testosterone (pg/mL)  (*n*=7/28) | 1.3 (0.6) | 3.6 (2.2) | 70.0 (30.1) | 69.5 (27.3) |
| Oestradiol (pg/mL)  (*n*=7) | 44.7 (42.6) | 41.6 (27.9) | - | - |
| Myoglobin (ng/mL)  (*n*=7/27) | 107.3 (72.1) | 415.6 (360.4) | 329.3 (405.5) | 622.3 (405.6) |
| MDA (pmol/mL)  (*n*=7/27) | 158.4 (69.4) | 131.5 (54.2) | 97.0 (48.9) | 112.1 (67.5) |
| TAC (mM)  (*n*=7/27) | 0.8 (0.1) | 0.6 (0.2) | 0.8 (0.3) | 0.8 (0.3) |
| COMP (pg/mL)  (*n*=7/27) | 2,576.9 (1,558.8) | 2,617.7 (1,944.3) | 2,085.7 (1,438.4) | 2,415.6 (2,124.2) |

**Table S5.** Pre- and post-race data for rowers, split by sex.

**Supplementary Information 6. Change in biomarkers by competition.**

1. Biomarkers of energetic stress


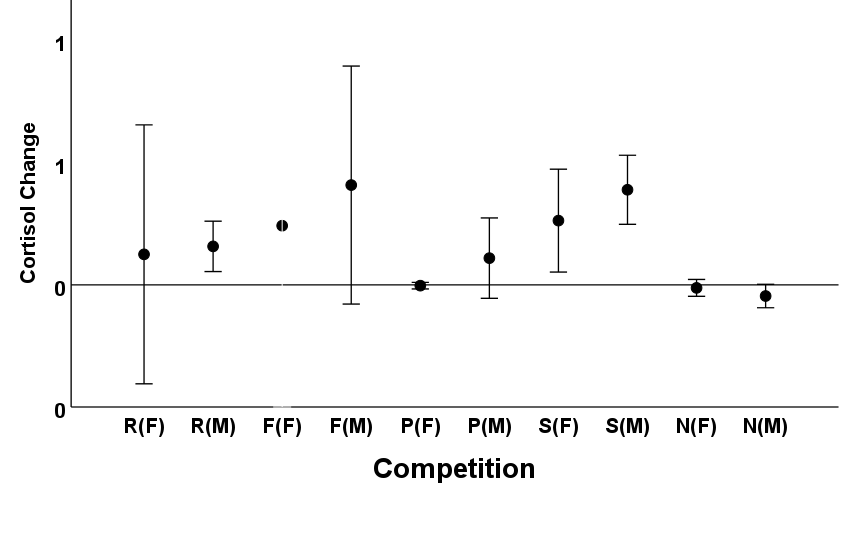


**
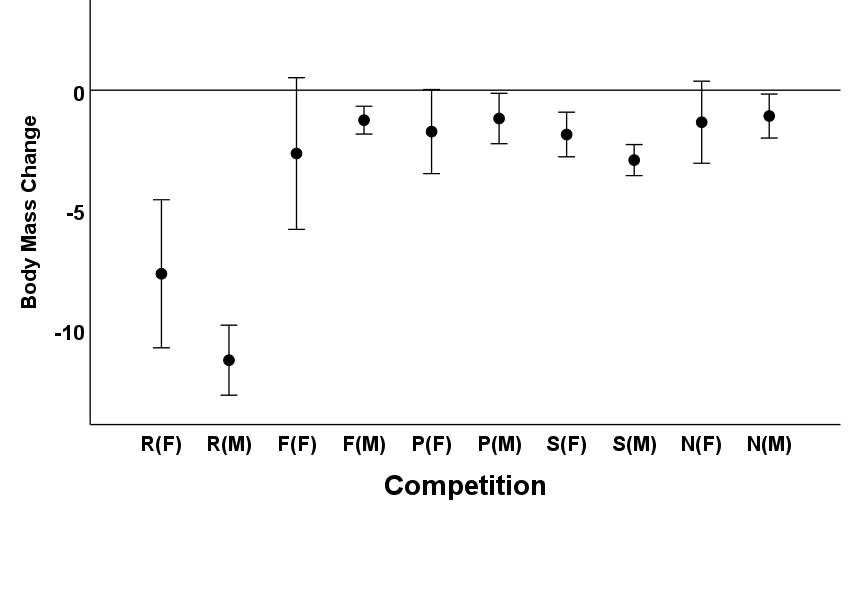
**

1. Biomarkers of defense


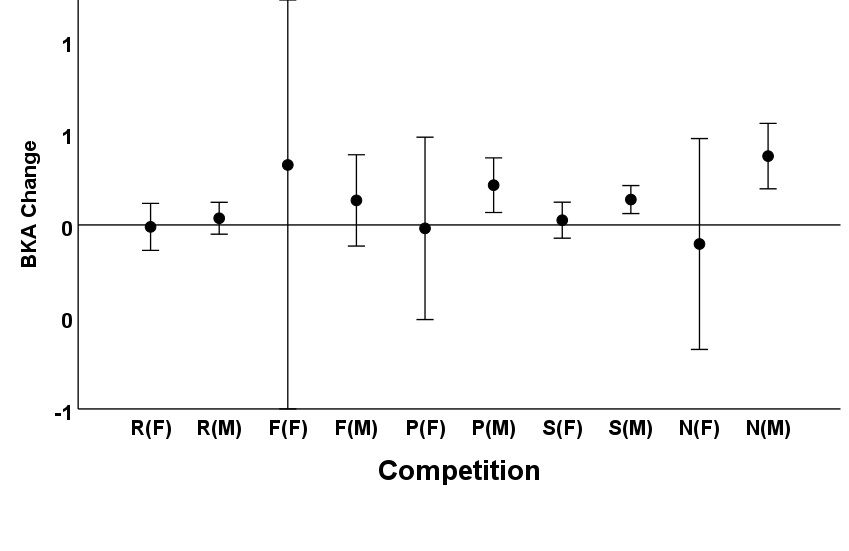


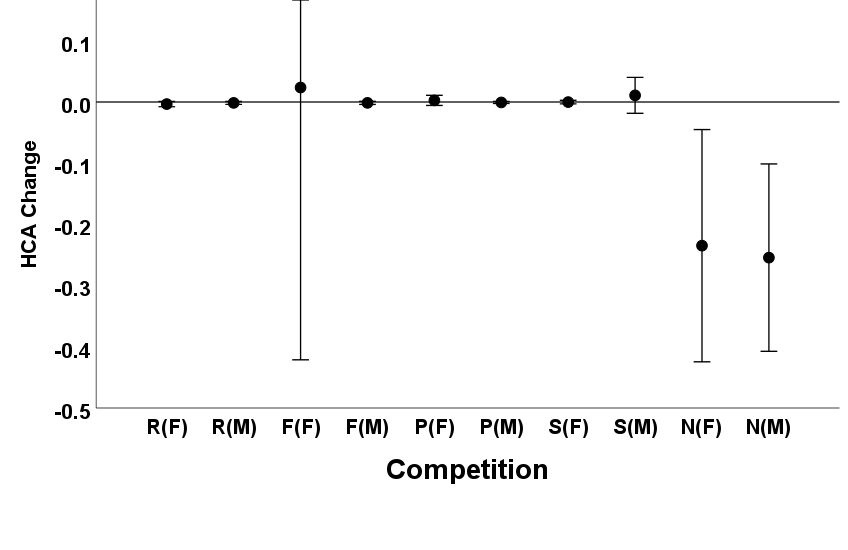

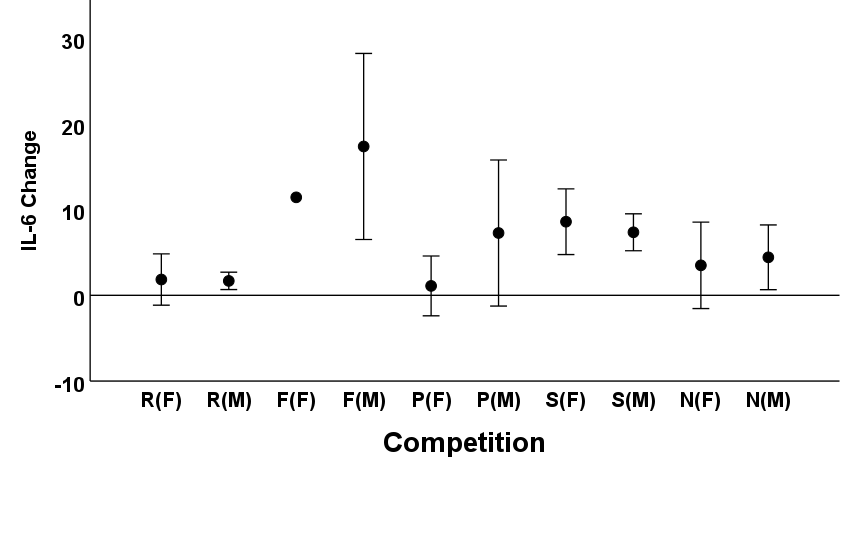


1. Biomarkers of storage


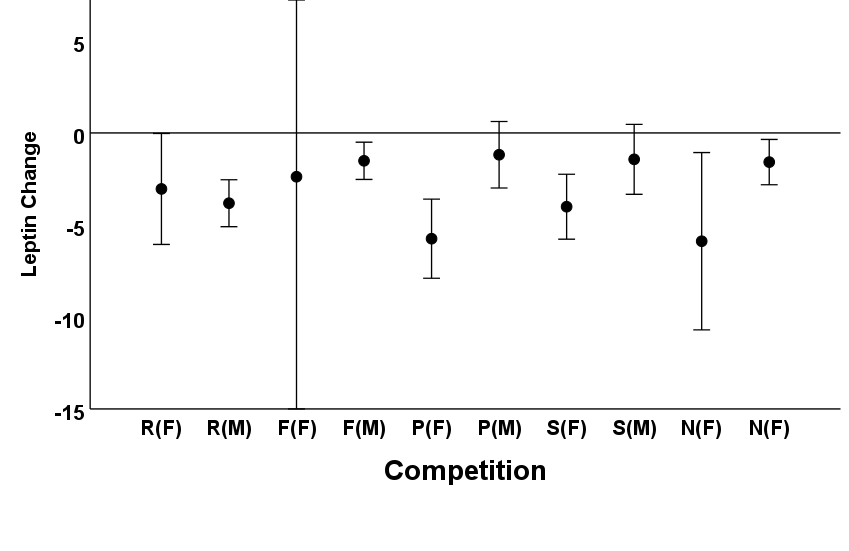

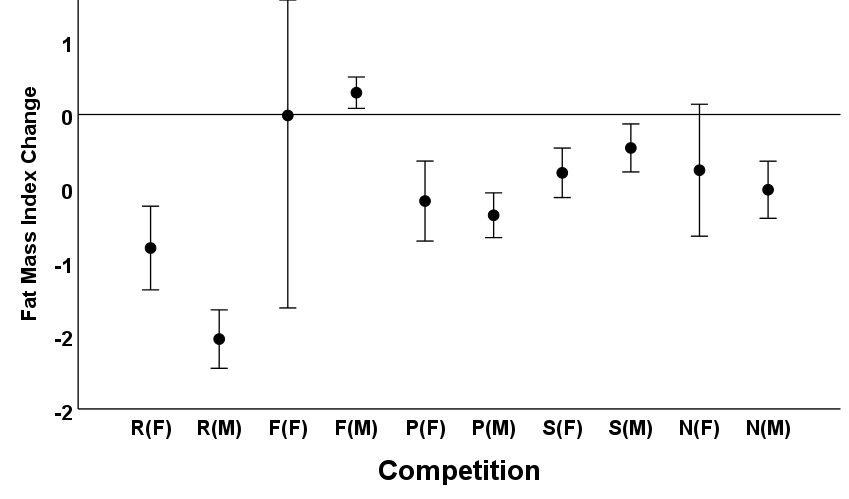


1. Biomarkers of reproduction


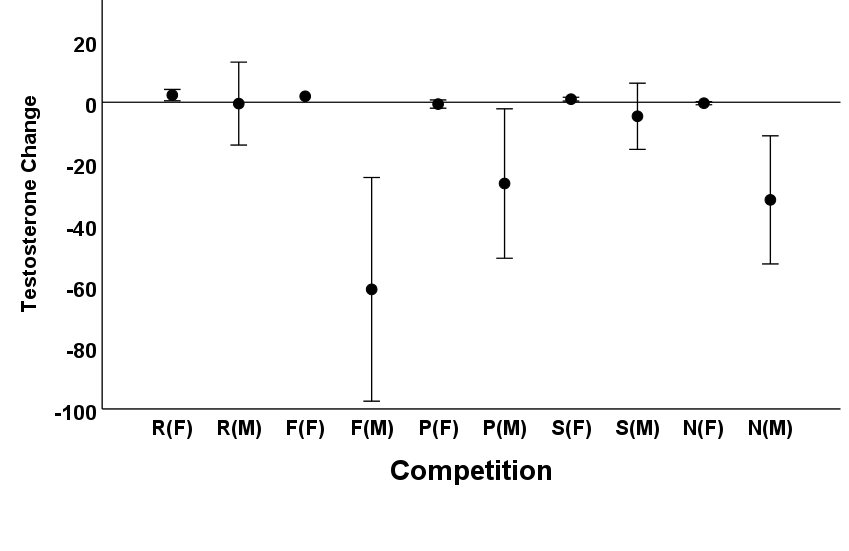

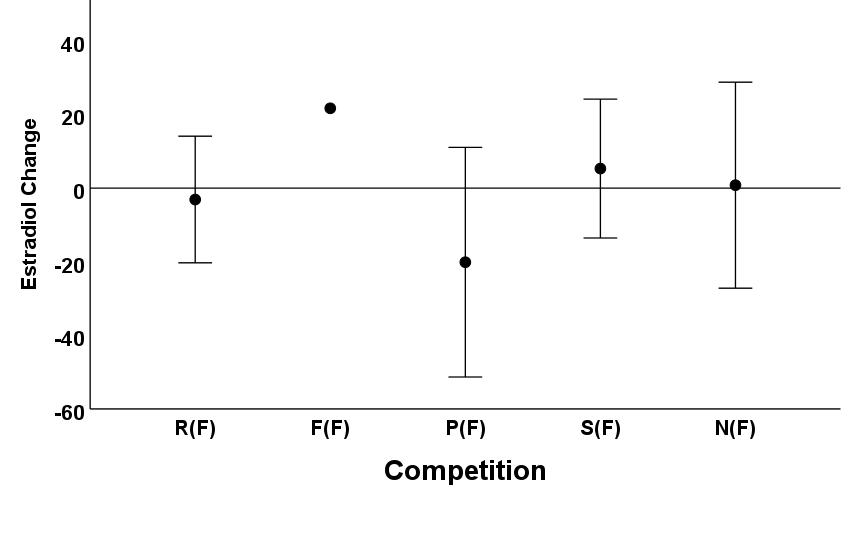


1. Biomarkers of maintenance


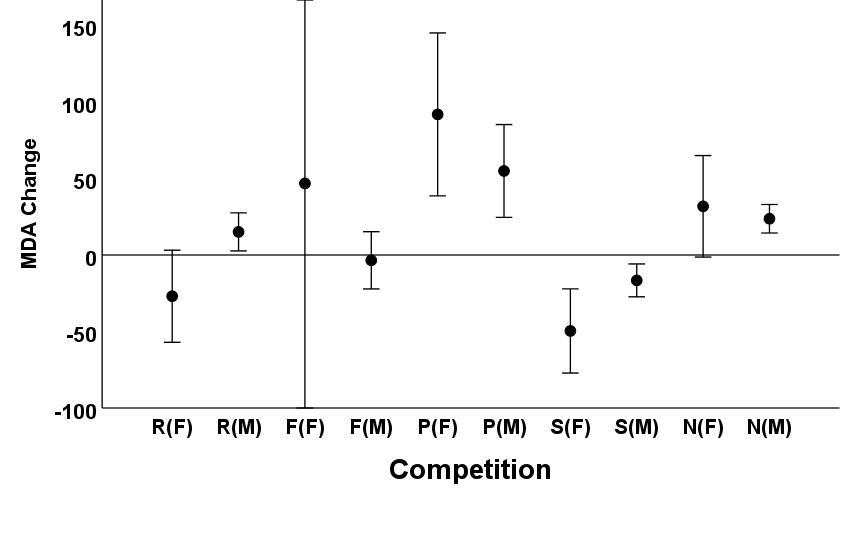


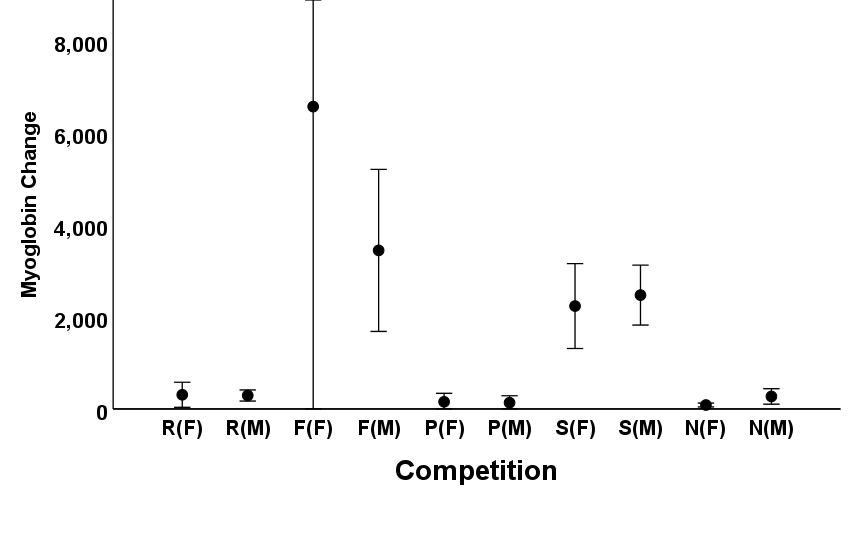


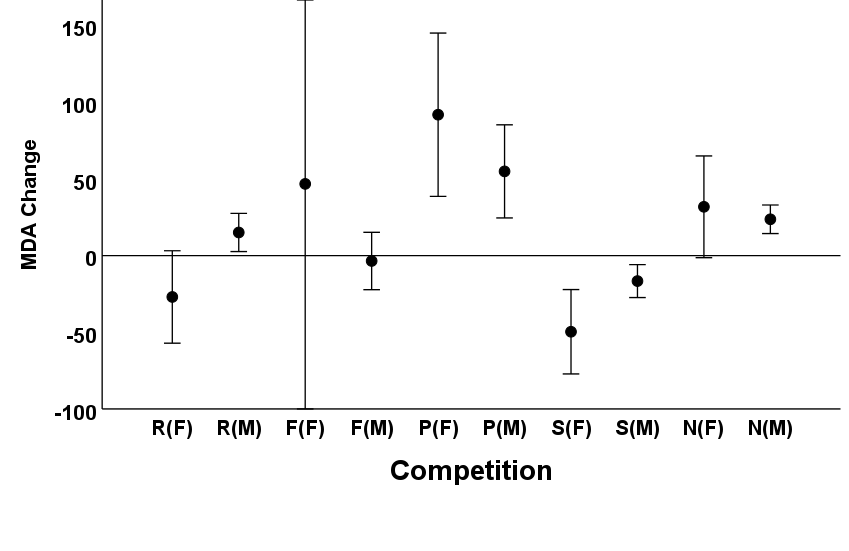

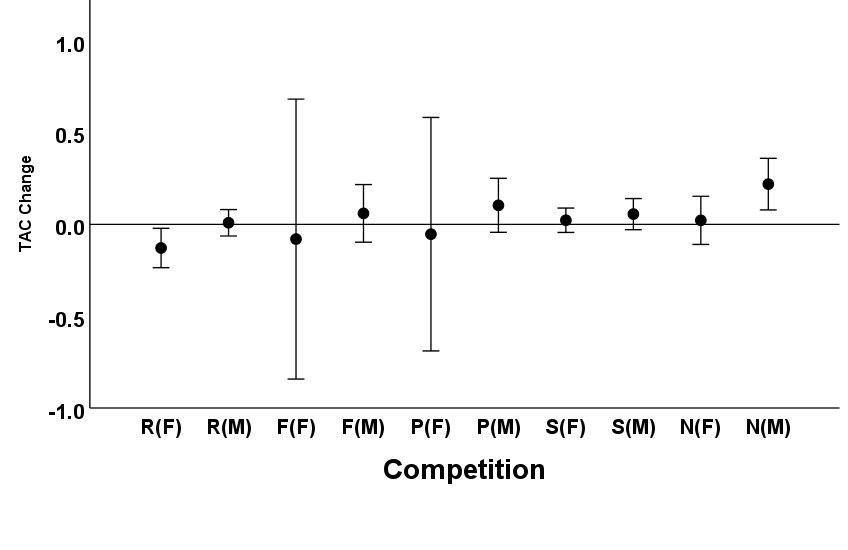


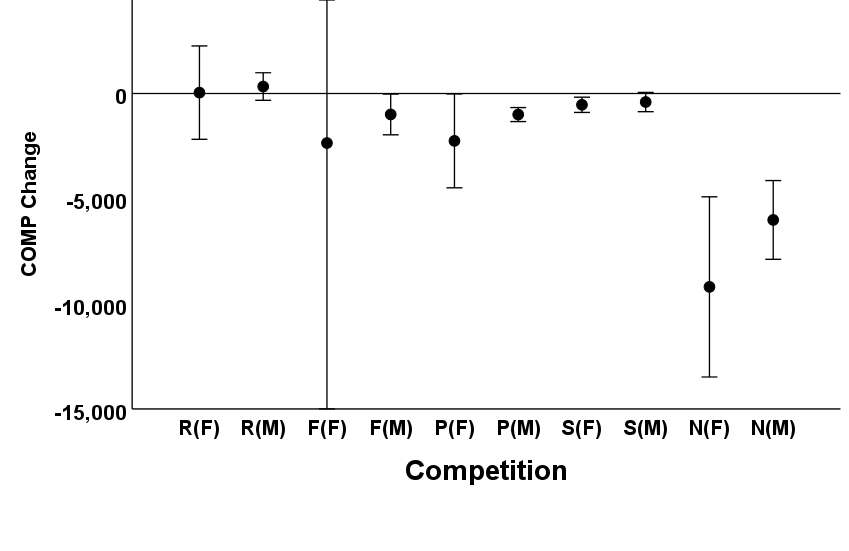


**Figure S6.** Graphs of biomarker change for each competition. R = Row, F = Finland, P = Peru, S = Spain and N = Nepal. The letter inside the bracket denotes sex (F = Female, M = Male). Error bars represent a 95% confidence interval of the mean change.
